# Preparation and Assessment of Heat-Treated α-Chitin Nanowhiskers Reinforced Poly(viny alcohol) Film for Packaging Application

**DOI:** 10.3390/ma11101883

**Published:** 2018-10-02

**Authors:** Chao Peng, Guangxue Chen

**Affiliations:** State Key Laboratory of Pulp and Paper Engineering, South China University of Technology, Guangzhou 510640, China; myaddresspc@163.com

**Keywords:** α-chitin nanowhiskers, heat treatment, thermal stability, barrier property, packaging application

## Abstract

In this study, poly(vinyl alcohol) (PVA) composite films enhanced by α-chitin nanowhiskers (ChWs) were prepared through heat treatment. The obtained membranes were assessed by means of FTIR spectroscopy, X-ray diffraction, thermogravimetric analysis, regular light transmittance, mechanical tests, permeability and water absorption. The influence of the nano-component and heat treatment on the mechanical, thermal and water-resistant properties of the composite membrane were analyzed. From the results of the work, the produced films with excellent barrier properties and inexpensive raw processed materials have great prospects in packaging applications.

## 1. Introduction

The requirement of fully taking advantage of more environmentally friendly barrier materials, in substitution of other unrecoverable polymer materials derived from petrochemical origin, is constantly orienting research activities towards the renewable biopolymers in many applications, especially in food packaging [1]. Among various environmentally and nontoxic barrier materials, poly(vinyl alcohol) (PVA) is a most significant semi-crystalline polymer. It has outstanding performance on solvent resistance, film-forming ability, transparency, non-toxicity, biocompatibility and oxygen barrier properties. Therefore, it has been widely used in various industrial applications, such as adhesives [2], packaging application [3], paper coating agents [4] and drug delivery carriers [5]. However, its actual utilizations are restricted by its poor resistance to water molecules, caused by hydroxyl groups in the repeating unit of PVA. For the sake of its excellent water resistance performance, PVA is typically blended with other synthetic polymers or else reinforced with different nanoscale materials such as clay [6] and grapheme [7,8]. In addition, a deal of researchers have concentrated on modifying its chemical properties by chemical cross-linking using compounds such as 1, 6-hexamethylene diisocyanate [9], glutaraldehyde [10], glyoxal [11], and boric acid (BA) [12]. Even though the treatments with chemicals may be convenient and simple, they elevate the certain risk of toxic materials. Many other green methods have also been developed including heat treatment [13] and ultra violet (UV) radiation [14].

Chitin with the units of N-acetyl-D-glucosamine, is the most abundant polysaccharide, second only to the cellulose [15]. Chitin consists of three crystalline forms of α-chitin, β -chitin, and γ-chitin, and the α-chitin mainly exists in the crustacean shells of shrimps and crabs. In the food industry, most of the shells of seafood are currently disposed of as rubbish. One effective methods to improve economic benefits is to manufacture high-performance chitin nanowhiskers. ChWs shows many advantages over traditional inorganic nanoparticles such as nontoxicity, easy modification, biocompatibility and biodegradability. The development of ChWs related applications is much slower than those of cellulose nanofiber [16]. However, ChWS still has important strategic significance in the period of resource shortage because of its rich availability and special performance. During the past decade, chitin nanowhiskers have been applied in a variety of synthetic polymers to achieve enhanced effects without compromising other properties such as transparency. Most recently, a research interest in chitin nanowhiskers for reinforcing PVA films has rapidly arisen. Sriupayo et al. [17] reported a α-Chitin nanowhisker enhanced poly(vinyl alcohol) nanocomposite film, which exhibits excellent mechanical properties. In this study, the process was to first blend the two solutions and then carried out the casting film. Thermal stability and tensile strength of α-chitin nanowhisker-reinforced PVA films strongly improved, compared with that of the unmixed PVA film having an initial increase in the nanowhisker content. However, Deng et al. [18] reported a new preparation method. They prepared and characterized a ChWs–PVA composite film by soaking pre-shape ChWs sheet into PVA solution. The results showed that the α-chitin nanowhisker was a crucial component for the process of increasing the thermal stability and mechanical properties of resulting films, which the Young’s modulus and tensile strength was up to 5.70 GPa and 127 MPa. Besides this, composite films of PVA with the chitin nanowhiskers were carried out via filtration of the dispersion by Kadokawa et al. [19]. According to their research, the chitin nanowhiskers were prepared by using an ionic liquid, which contained 1-allyl-3 methylimidazolium bromide (AMIMBr). There are several works related to α-chitin nanowhiskers/PVA composite films, but the further improvement of barrier performance of composite membrane by a green and convenient modification method has hardly been investigated, especially in packaging applications. Therefore, it is valuable to provide a renewable and high added-value film with great stability and barrier property for industrial applications. 

In this study, α-chitin nanowhiskers (ChWs) suspension was prepared from being partially deacetylated by shrimp shells. The composite films of the α-chitin nanowhiskers with PVA were obtained via immersing ChWs films into PVA solution. The aim was to evaluate the reinforcement effect of ChWs and heat treatment to barrier properties of the PVA/ChWs composite membrane. The chemical components of ChWs, PVA, and PVA/ChWs membrane were identified by an X-ray diffractometer (XRD) and a Fourier transform infrared spectroscopy (FTIR). The thermal, optical, tensile strength of ChWs films, PVA films, and PVA/ChWs composite films were tested and analyzed. The oxygen barrier properties and moisture barrier properties of the resulting composite film with or without heat treatment were also investigated.

## 2. Materials and Methods

The shrimp and crab shell flakes were purchased from Shanghai Macklin Biochemical Co., Ltd. (Shanghai, China). Poly (vinyl) alcohol (PVA-26, degree of alcoholysis: 97.0–98.8) was obtained from Aladdin Ltd. (Shanghai, China). Sodium hydroxide (NaOH), hydrochloric acid (HCl), sodium hypochlorite (NaClO), sodium chlorite (NaClO_2_), and sodium borohydride (NaBH_4_) were obtained from Shanghai Macklin Biochemical Co., Ltd. All other chemicals were of analytical grade and were used without further purification.

### 2.1. Preparation of α-Chitin Nanowhiskers (ChWs) Suspension

The confirmed method for refining in the previous studies was used [20], with minor modifications. In brief, 25 g raw waste shrimp and crab shell flakes were suspended in 400 mL of 1 M NaOH solution by stirring at 80 °C for 3 h to eliminate the remaining proteins. After washing fully to neutral, the resulting wet lump dried in a vacuum drying oven overnight, then performed the same operation after the 1 M HCl solution treatment. After that, the sample was then bleached with 0.3% w/v NaClO_2_ at 25 °C for 1–2 h. After bleaching, the extracted sample was partly deacetylated in 33 wt% NaOH solution with adding 0.03 g of NaBH_4_ by stirring at 90 °C for 4 h. The resulting wet chitin cake was then mixed with 400 mL deionized water and controlled the pH value to 3.5 with 1 M acetic acid while stirring. Finally, the transparent partially deacetylated ChWs suspension was preserved at −4 °C.

### 2.2. Fabrication of PVA/ChWs Composite Film

A solution impregnation method was used to prepare the composite films. After air bubbles were removed from the suspension by centrifugation for 10 min at 5 °C, a transparent ChWs suspension with the concentration of 0.1 wt% was achieved. The resulting ChWs suspension was firstly cast on a polytetrafluoroethylene (PTFE) plate with a 40 ± 5 μm thick layer, and dried at 55 °C by humidity chamber under mild pressure for 48 h. After that, a pure ChWs film was obtained. Next to it, a 10 wt% aqueous solution of PVA powder first swelled in deionized water at room temperature by magnetic stirring for half an hour, and then raised to 85 °C until completely dissolved. After cooling the PVA aqueous solution to room temperature, the ChWs film was impregnated in the PVA aqueous solution for the night. The PVA-impregnated fibrous mats first were air-dried at room temperature overnight, and followed by a 12 h-dried at 40 °C to obtain a composite film, which came into being a sandwich-like structure of PVA-ChWs-PVA. Finally, the obtained membranes were heat treated by high temperature sterilizing oven at 120 °C for 5 min to restrain their solubility in water. The neat PVA films were also prepared under the same conditions and used as control groups.

## 3. Characterization

### 3.1. Fourier Transform Infrared Spectroscopy (FTIR)

FTIR spectra of PVA solid powder, pure ChWs film, PVA/ChWs composite membrane and heat treated PVA/ChWs composite membrane were recorded via a spectrophotometer (VERTEX 70, Bruker, Germany) with ATR mode. The dried sample materials were ground together with infrared grade KBr to prepare the experimental translucent disks and then compressed. The FTIR spectrums were analyzed by recording 32 scans between 4000 and 500 cm^−1^ with a resolution of 4 cm^−1^.

### 3.2. X-ray Diffraction (XRD)

The chitin powder, neat ChWs film, pure PVA film, PVA/ChWs film and heat treated PVA/ChWs composite film were characterized by a polycrystal X-ray diffraction at a range of 2θ= 10–60° (D8 ADVANCE, Bruker, Germany).

### 3.3. Thermal Gravimetric Analysis (TGA)

The thermal decomposition behavior of the membranes of pure ChWs film, pure PVA film, and PVA/ChWs film was investigated by a Q500 TGA System (TA Instruments, New Castle, DE, USA) with a temperature range from 40 °C to 500 °C at a heating rate of 5 °C/min under the N_2_ atmosphere.

### 3.4. Mechanical Properties

The tensile mechanical properties of the samples were investigated by an electronic universal material-testing machine (INSTRON 5565, Instron Corp., Canton, MA, USA) at room temperature. The samples were cut into 20 mm long, 10 mm wide with a crosshead speed of 1 mm/min. The pure PVA films and ChWs films were also tested under the same conditions for used as control groups. Tensile strength and Young’s modulus were calculated in triplicate.

### 3.5. Light Transmittance

A UV–visible spectrometer (Cary60, Agilent, Santa Clara, CA, USA) was applied to test the regular light transmittance of ChWs film, PVA film, PVA/ChWs composite film and heat treated PVA/ChWs composite film. The wavelength range is 200–1000 nm with a speed of 600 nm/min.

### 3.6. The Oxygen Transmission Rate (OTR)

A Gas Transmission Rate Tester (VAC-V1, Labthink, Jinan, China) was applied to check out the oxygen transmission rate (OTR). The samples were cut into rounds with 100 mm diameter. Before testing, the samples should be placed in the dryer for at least 48 h and the convex edge was coated with a layer of grease to achieve the sealing effect. Then the sample was carefully put in to keep the samples neat and clean. N_2_ was used to blow up the air in the cavity until the voltage was stable. OTR was measured under certain humidity and temperature (50% relative humidity and 23 °C). All samples were taken in triplicate and finally averaged.

### 3.7. The Water-Resistance Pressure (mm) Tests

The water-resistance pressure (mm) tests of all the samples were actualized based on a conventional technique [21]. Each of sample was made into round piece with a diameter of 30 mm and then taken them to block one end of a graduated thin tube with a diameter of 8 mm. Then the thin tube was added drop-wise with deionized water until the sample was permeated by water, the height of the water column was taken down. All samples were taken in triplicate and finally averaged.

## 4. Results

### 4.1. Fourier Transform Infrared Spectroscopy (FT-IR)

**Figure 1:**
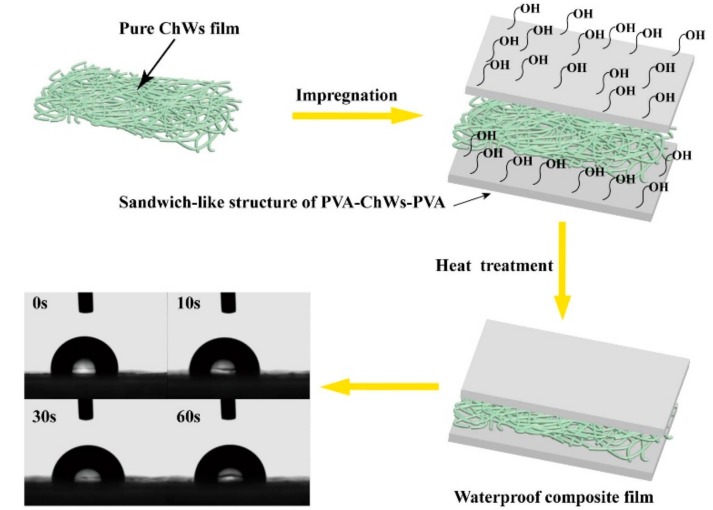
Flow chart of the preparation of PVA/ChWs composite film.

**Figure 2:**
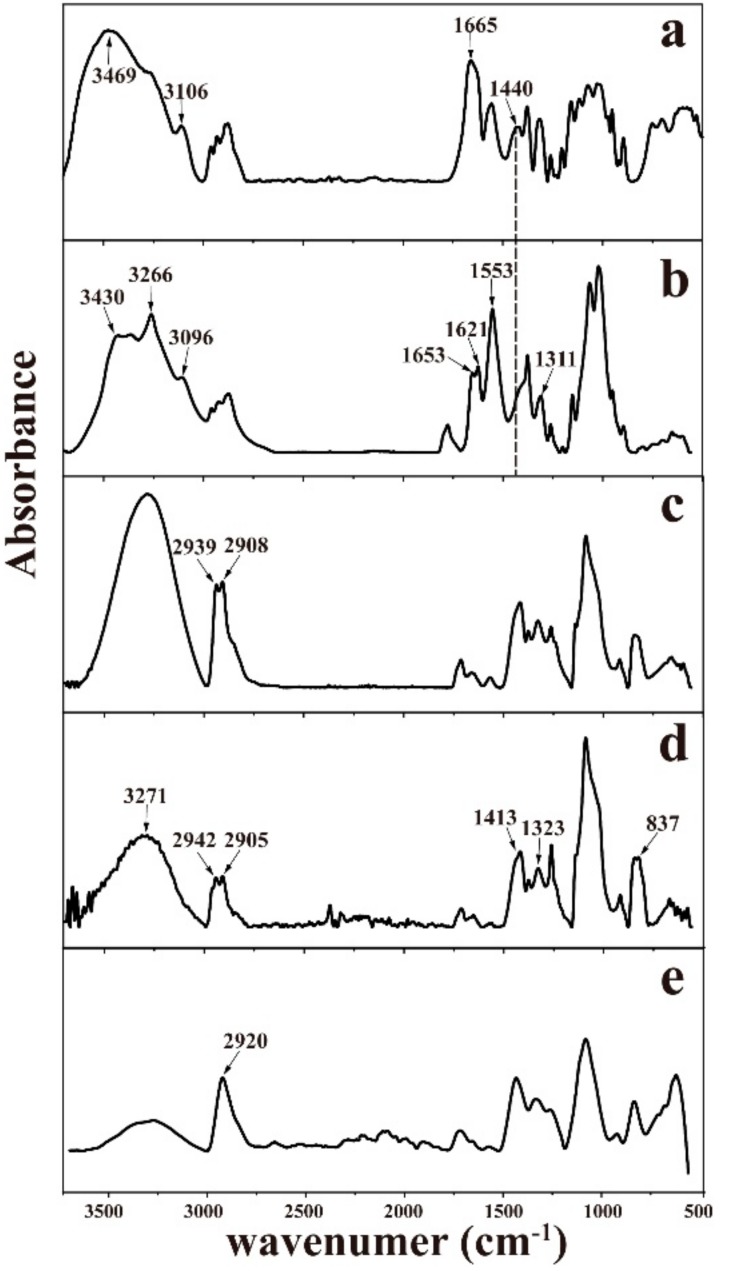
FTIR spectrum of the chitin powder (**a**), ChWs film (**b**), PVA/ChWs composite films (**c**), pure PVA films (**d**) and heat treated PVA/ChWs composite films (**e**).

Figure 1 was a flow chart of the preparation process of PVA/ChWs composite membrane. The obtained membranes were then assessed by means of FTIR spectroscopy. And Figure 2 illustrated FT-IR spectra over the wave number range of 4000–500 cm^−1^ of the chitin powder, ChWs film, PVA/ChWs composite films, pure PVA films and heat treated PVA/ChWs composite films. According to Figure 2a, the absorption peak at 1440 cm^−1^ representing proteins was obvious in the spectra of the chitin powder. While in Figure 2b, it disappeared, proving the raw chitin powder was fully purified via alkali treatment. According to Figure 2b, the characteristic peaks at 1653 cm^−1^ and 1621 cm^−1^ (Amide I), 1553 cm^−1^ (Amide II), and 1311 cm^−1^ (Amide III) were obvious, which represented the α-chitin nanowhisker. The peaks at 3430 cm^−1^, 3266 cm^−1^, and 3096 cm^−1^ were also shown in Figure 2b, which represented the stretching of intramolecular and intermolecular OH and CH_2_OH vibrations, stretching of NH_2_, and NH secondary amides vibrations, respectively. The characteristic amide I (–CONH–) peak at 1653 cm^−1^ was obvious in the spectra of PVA/ChWs composite films (Figure 2c) and heat treated PVA/ChWs composite films (Figure 2e). The characteristic peaks of PVA can be found in Figure 2d, including the peaks at 837 cm^−1^ (CH_2_ rocking), 1323 cm^−1^ (OH with CH wagging), 1413 cm^−1^ (OH, CH bending), 2905 cm^−1^ (symmetric stretching of CH_2_), and 2942 cm^−1^ (asymmetric stretching of CH_2_)*.* And the spectrum of PVA/ChWs composite film (Figure 2c) was very similar to this of PVA with slight migration. This was because the PVA layer was at the top of the sandwich-like structure. The signals just reflected the PVA surface layer of the PVA-ChWs-PVA film under the ATR mode of the FTIR measurement [21]. In addition, no other new peaks were found in the spectrum of the composite film, surmising the impregnation with forming a sandwich-like structure was just a physical process without chemical reaction. 

### 4.2. X-ray Diffraction (XRD)

**Figure 3:**
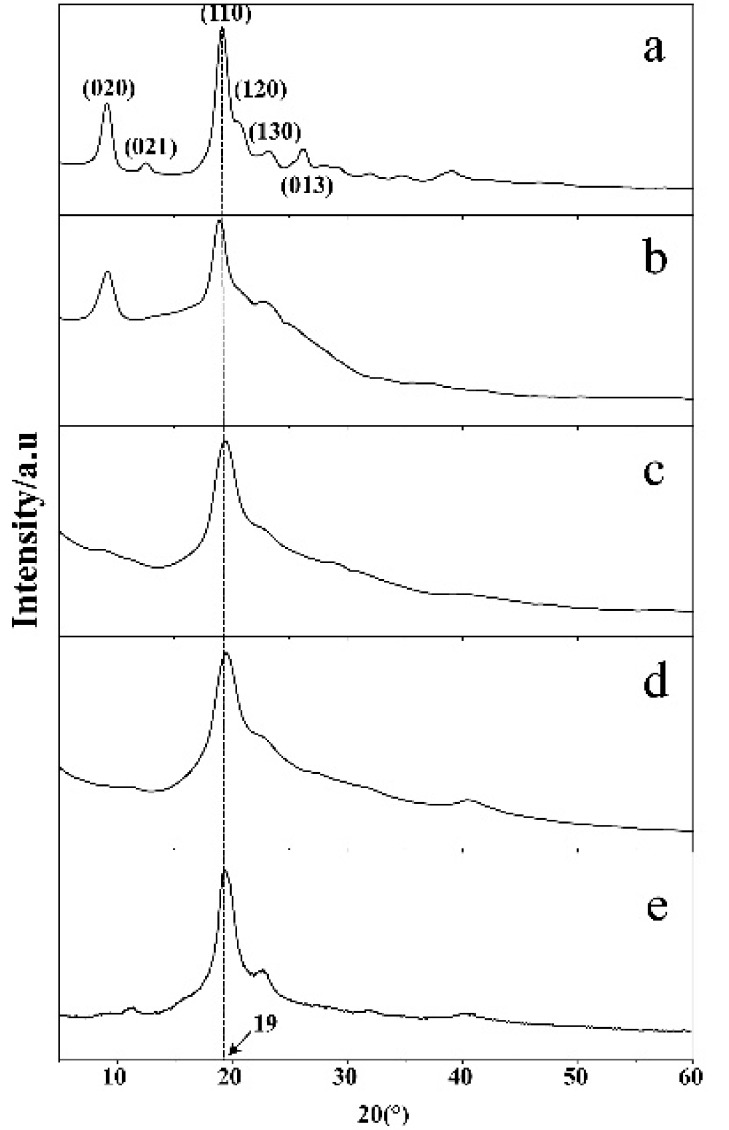
Wide-angle X-ray diffraction patterns of the chitin powder (**a**), ChWs film (**b**), PVA/ChWs composite films (**c**), pure PVA films (**d**) and heat treated PVA/ChWs composite films (**e**).

By X-ray diffraction of the component, the diffraction pattern was investigated to gain information about the composition of PVA/ChWs the structure or morphology of the atoms or molecules inside resulting membrane. The diffraction peaks of chitin powder were appeared at 9°, 12.6°, 19°, 21°, 23.4°, and 27.1° in Figure 2a, which were indexed as the (020), (021), (110), (120), (130), and (013), respectively [22]. For partially deacetylated α-chitin nanowhiskers, their X-ray diffraction pattern exhibited two typical reflections of pure α-chitin peaks at 2θ angles of about 9° and 19°, which indicated that crystallinity and morphology were maintained after partially deacetylated. By comparing X-ray diffraction pattern of pure PVA membranes (Figure 3d) and the composite membranes (Figure 3c), the X-ray diffraction patterns of these two were very close. The diffraction pattern also proved that the crystalline structure of PVA was not affected by the incorporation with α-chitin nanowhiskers.

### 4.3. Thermal Stability

**Figure 4:**
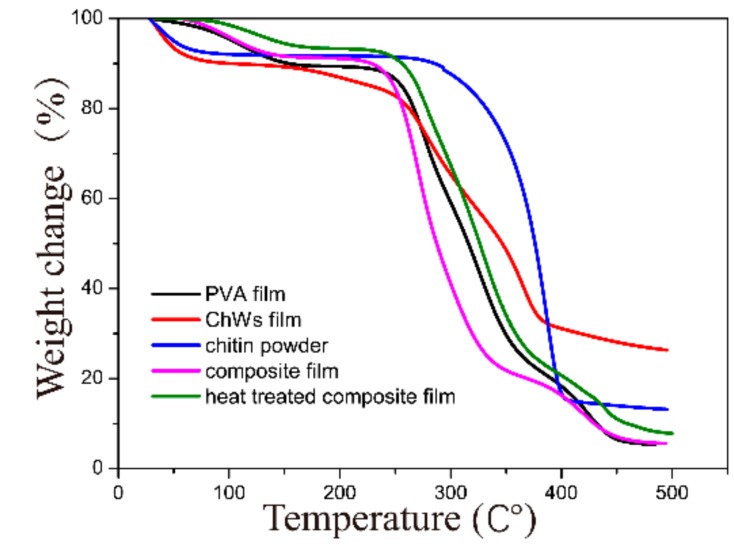
TGA curves of the chitin powder, ChWs film, PVA/ChWs composite film, the pure PVA film and heat treated PVA/ChWs composite film.

In many industrial applications including food packaging, barrier materials are required for appropriate thermal stability to tolerate harsh temperature and humidity changeable activities. Figure 4 showed TGA thermograms of the raw chitin powder, pure ChWs film, PVA/ChWs composite film, the pure PVA film and heat treated PVA/ChWs composite films, respectively. All the samples investigated showed three main weight loss stage. The first weight loss area was at about 50–90 °C, likely a result of the evaporation of physically weak and chemically strong bound water [23]. The second transition region was at 110–270 °C. On the basis that the TGA curves, the PVA membrane performed a primary degradation point at 230 °C and chitin powder began to disintegrate at 268 °C, while the ChWs film showed this of at 159 °C. According to Figure 4, the primary degradation peaks for composite membrane were quite similar to those of the single component membrane. It can be determined that the thermal decomposition of the PVA/ChWs composite films provided a shift toward higher temperature after heating, suggesting an anti-plasticizing effect. The heat treatment of the PVA/ChWs composite film perhaps performed a vital effect on the thermal deformation. 

### 4.4. Tensile Properties

**Figure 5:**
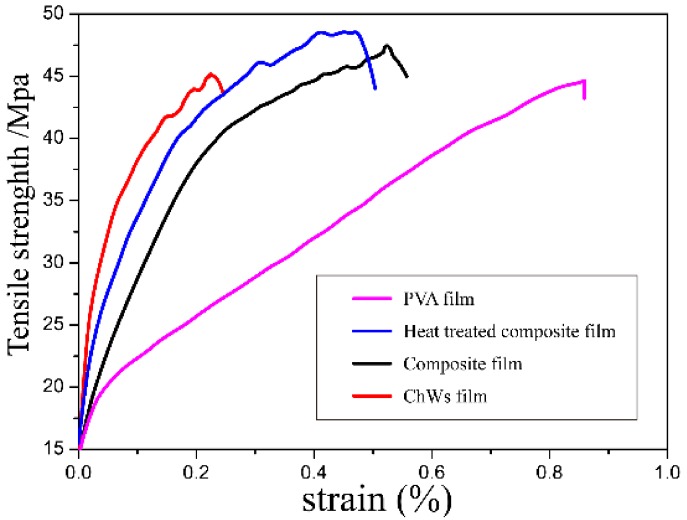
Tensile strength of the pure PVA film, ChWs film, PVA/ChWs composite film and heat treated PVA/ChWs composite film.

Figure 5 showed the tensile strength of the pure PVA film, ChWs film, PVA/ChWs composite film and heat treated PVA/ChWs composite film. The Young modulus of pure PVA film was 207.41 MPa, where the tensile properties of composite membrane had a big boost, due to the interface created between polymer and nanowhiskers and between nanowhiskers themselves [24]. Besides, the Young modulus of the heat treated PVA/ChWs membranes showed the higher value (311.23 MPa) than PVA/ChWs composite film. Heat treatment had been used to composite film to cut down hydroxyl group on the surface of the membrane (as shown in Figure 1) for the applications area where a waterproof property was required. The heat treatment on outer PVA layer also assisted in raising their appearance stability and mechanical properties, due to an increase in the crystalline fraction of the polymer. In addition, heat treatment may make the composite film form more physical cross-linking points between ChWs and PVA. However, the mechanical properties enhancement resulted in the elongation breaking as a sacrifice. The elongation break point value of two composite films were 46%, 51%, respectively, which is much shorter than pure PVA film (85.4%). 

### 4.5. Regular Light Transmittance

**Figure 6:**
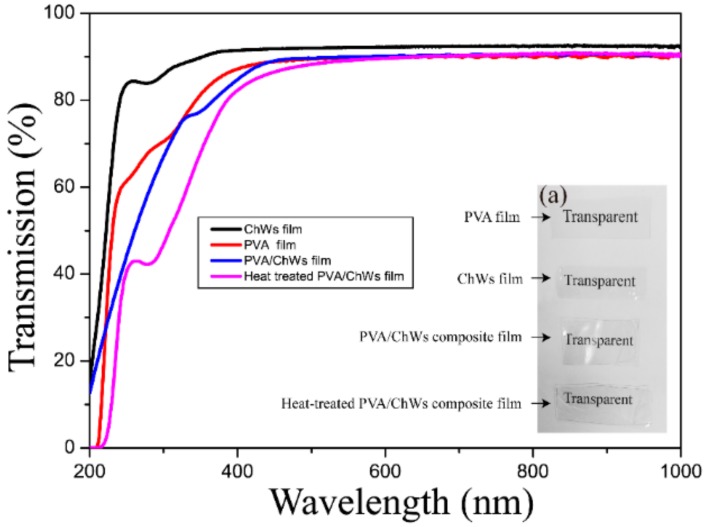
(a) Photographic images and UV–vis spectra of the pure PVA film, ChWs film, PVA/ChWs composite film and heat treated PVA/ChWs composite film.

Transparency of materials is also required in many applications, especially in packaging applications. The light transmittance spectra of the pure PVA film, pure ChWs film, PVA/ChWs membrane and heat treated PVA/ChWs composite membrane were shown in Figure 6. At a visible wavelength of 600 nm, the visible light transmittance of all films was up to 90%, and the visible light transmittance of the ChWs membrane was even up to 93%, which may be because light can pass directly through the cavity of the α-chitin nanowhiskers. Meanwhile, although the composite film had been heat treated, the transparency was not completely affected, which is clearly shown in Figure 6a as the text behind both of the composite films looked clear.

### 4.6. Water Resistance and Barrier Properties

**Figure 7:**
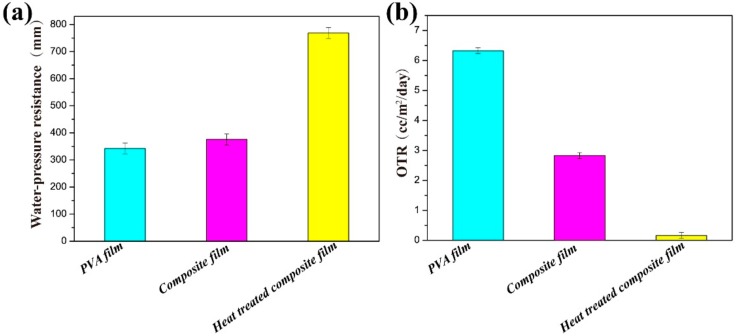
Water-resistant pressures (**a**) and OTRs (**b**) of the pure PVA film, PVA/ChWs composite film and heat treated PVA/ChWs composite film.

**Figure 8:**
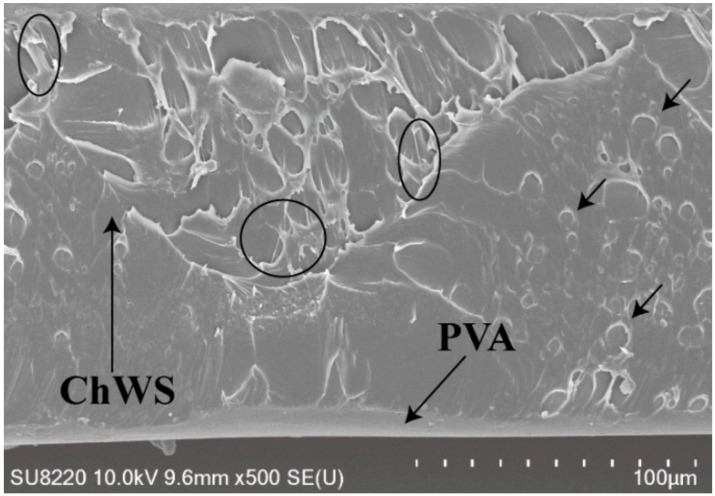
Fractography of heat treated PVA/ChWs composite film after cutting by ion beam.

Packaging materials require good barrier properties to provide product protection against environmental damage and prolong long-term performance [25]. Regarding to the PVA/ChWs composite films, water resistance properties and oxygen barrier properties were tested and analyzed in detail. The results were shown in Figure 7a,b. The water-resistant pressure of the pure PVA films was raised substantially (768.72 mm) by collaborating with α-chitin nanowhiskers films via heat treatment, demonstrating that this technology was a very forceful way to enhance the water resistance of pure PVA films. Meanwhile, the pure PVA film had a very low oxygen permeability (6.32 cc/m^2^/day) as a result of its crystalline structure and strong interaction between molecules [26], then the oxygen transmission rate of the composite film declined from 2.82 to 0.16 cc/m^2^/day before and after heat treatment. In addition, contact angle testing enables this performance to be seen more intuitively. The shape of the water-drop on the surface after heat treatment had a contact angle of 99.5° ± 2° (Figure 1) with few changes in shape within 60 s. According to fractography of heat treated PVA/ChWs composite film after cutting by ion beam (Figure 8), some thin fiber torn out over the matrix (indicated by circles). In addition, the ChWs layer with a rough surface was revealed to distribute with small round bump and some cavities. PVA solution was completely impregnated in the cavity of ChWS layer, which may be the reason for the formation of circular bumps. Then, the existence of these cavities may bring about longer and more tortuous pathways for the gas and water molecules, thereby improving the barrier properties. Therefore, the improvement of barrier performance may be due to two factors, one was the variations in morphologies and the chemical structure in membranes [27] caused by heat treatment, and the other was the generation of the zigzag pathways for the gas and water molecules [28]. 

## 5. Conclusions

Nanocomposites based on poly(vinyl alcohol) matrix enhanced with partially deacetylated α-chitin nanowhiskers (ChWs) were obtained by impregnating a dried ChWs film in the homogeneous PVA solution and finally heat treating. Thermal, optical, mechanical properties, barrier and water resistance properties, as well as the chemical components of the resulting composite materials were investigated. Incorporation of α-chitin nanowhiskers resulted in raising the thermal stability of the resulting composite membranes, while there was no side influence on the crystalline structure of the PVA. The mechanical properties of the composite membrane were also enhanced, while the percentage of elongation at break was sacrificed. By combinatorial application with α-chitin nanowhiskers and subsequent heat treatment, the resulting composite film displayed an excellent ability to obstruct oxygen and water, making membrane more stable even in moist environments, which is important for packaging applications.
